# Sonographic Detection of Abdominal Free Fluid: Emergency Residents vs Radiology Residents

**DOI:** 10.5812/traumamon.5476

**Published:** 2013-01-15

**Authors:** Majid Shojaee, Gholamreza Faridaalaee, Anita Sabzghabaei, Saeed Safari, Hamid Mansoorifar, Ali Arhamidolatabadi, Fatemeh Keyghobadi

**Affiliations:** 1 Emergency Medicine Department, Imam Hossein Hospital, Shahid Beheshti University of Medical Sciences, Tehran, IR Iran; 2 Emergency Medicine Department, Shohada Tajrish Hospital, Shahid Beheshti University of Medical Sciences, Tehran, IR Iran; 3 Emergency Medicine Department, Alzahra Hospital, Tabriz University of Medical Sciences, Tabriz, IR Iran

**Keywords:** Ultrasonography, Trauma, Emergencies, Abdominal Injuries

## Abstract

**Background:**

Focused assessment with sonography for trauma (FAST) has become a part of initial examinations in trauma care at emergency departments (ED).

**Objectives:**

The goal of the present study was to evaluate the accuracy of FASTs performed by emergency residents (ER) in detection of abdominal free fluid following blunt trauma.

**Materials and Methods:**

In this study, the reports of ERs performing FASTs on 286 admitted patients following blunt trauma were compared with those of radiology residents (RR) in relation to presence of abdominal free fluid. In addition, the reports of the two resident groups were compared with the final abdominal outcome, based on the results of abdominal computed tomography (CT) and clinical follow up.

**Results:**

The ERs had reported abdominal free fluid in 20 (6.9%) patients while RRs performing FAST had positive results in 22 (7.6%) patients. The reports of FASTs revealed significant correlation between the two resident groups (P < 0.001). ERs performing FASTs had 90% sensitivity and 98.5% specificity in comparison to RRs sonography reports. Furthermore, ER-performed FASTs had 96.5% accuracy in relation to final outcome.

**Conclusions:**

Following training, ED residents can perform FAST with high accuracy and specificity, similar to RR residents, in patients with blunt abdominal trauma.

## 1. Background

Since 1980 FAST has been a part of initial examinations and an invaluable adjunct in emergency care of patients with blunt abdominal trauma (BAT) (1). Over the past years, the use of FAST has increased due to its advantages of being non‐invasive, rapidly performed, and readily repeatable (2). Different studies have confirmed the upgrading of trauma care while FAST is included in the management of BAT (3, 4). At present, abdominal sonography is applied in blunt and penetrating trauma algorithms as an initial evaluation method in detection of abdominal free fluid. It has gradually been taken out of the radiologist monopoly, evolving to a common tool in a variety of specialties. In 2008, the American College of Emergency Physicians formalized recommendations for training of emergency physicians in FAST (5). Different studies have demonstrated a sensitivity of 42-96% and specificity of 85-100% for non-radiologists performing FAST (6, 7). Brenchley and their colleagues showed that emergency physicians can use FAST with sufficient specificity following training courses (8).

## 2.Objectives

This study aimed to evaluate the accuracy of FAST performed by ERs in detection of abdominal free fluid in patients admitted to the ED following BAT.

## 3. Materials and Methods

In this descriptive cross-sectional double-blind study, the reports of FASTs performed by ERs on patients, admitted to Imam Hossein Educational Hospital (Tehran, Iran) from April 2010 to March 2011, were compared with those carried out by radiology residents in relation to presence of abdominal free fluid. Firstly, the patients were assessed clinically by ERs and underwent FAST during primary or secondary trauma survey. Then, they were transferred to the Radiology Department within one hour of admission and re-evaluated by FAST performed by an RR. Abdominal computed tomography (CT) was carried out in positive or suspected reports of each resident group to confirm the diagnosis. In addition, patients with negative FAST results were observed for 6-12 hours in ED; in the absence of abdominal pain and tenderness they were discharged and followed-up by phone. Finally, data collected from sonography reports of the two resident groups were statistically compared for abdominal free fluid, using SPSS version 18 and chi-square test. Moreover, the correlation of the reports of two residency groups was evaluated using Pearson’s correlation test. Furthermore, the reports of the two resident groups were compared with the final abdominal outcome, based on the results of abdominal CT scans and clinical follow-up. The ERs had passed the theoretical and practical training courses of FAST by performing abdominal sonography on at least 120 patients under the supervision of an expert. Patients with unstable hemodynamics, penetrating trauma, age < 18 years and BMI > 30 were excluded.

## 4. Results

A total of 286 patients with BAT were evaluated during the study period (67% male). The ERs had reported abdominal free fluid for 20 (6.9%) patients while FAST reports performed by RRs were positive for 22 (7.6%) patients. For 59 cases with positive or suspected results of sonography, abdominal CT was performed. Only 14 (23.7%) cases had positive findings on abdominal CT scans. A total of 226 cases with negative FAST results based on reports of the two resident groups did not have any problems during the observation and follow-up periods. The reports of FASTs were significantly correlated between the two resident groups (r: 0.84, P < 0.001). ER-performed FASTs had 90% sensitivity and 98.5% specificity in comparison to sonography reports by RRs. Table 1 reveals the sensitivity, specificity and likelihood ratio of FASTs performed by the two groups in comparison to the final outcome based on findings of abdominal CT scans and clinical follow-up.

**Table 1.** Sensitivity, Specificity and Likelihood Ratio of FAST Performed by Two Resident Groups

Performance	Sensitivity	Specificity	Likelihood ratio	Accuracy
**Emergency resident**	60%	99.2%	127.8	96.5%
**Radiology resident**	59.1%	99.6%	68.8	96.5%

## 5. Discussion

The results of the current study showed that ER-performed FASTs had acceptable sensitivity and specificity in comparison to RR-performed sonography. In addition, ERs’ abdominal sonography was accurate in over 95% of cases in comparison to the final abdominal outcome, based on the results of abdominal CT scans and clinical follow-up. Rapid detection of abdominal complications following BAT in order to render appropriate emergency care can reduce mortality and improve outcome of trauma patients (9, 10). Application of FAST in ED could potentially provide critical information and optimize triage and transport of patients with multiple injuries. In previously published studies the sensitivity of FAST ranges from 75% to 100%, with specificity range from 88% to 100% (11). The necessity of presenting instructional items in relation to ultrasound scans and interpretation of the related data in the curriculum of emergency medicine specialists has been emphasized in a study carried out by Heller et al. (12). Emergency physicians with training can interpret sonography with relatively high sensitivity, specificity and accuracy in both pediatric and adult patients with BAT (13, 14). McKenney et al. evaluated the accuracy of 112 cases of FAST performed by surgical residents and reported that if the residents take the training courses, they can perform this test well for trauma patients (15). In other studies, non-radiologist specialists performed FAST with the sensitivity of 42-96%, specificity of 85-100% and overall accuracy of 89-99% (6, 7). The present study confirms these findings in relation to ER-performed FASTs. Following training, emergency medicine residents were able to perform FAST with high accuracy for patients with BAT. The relatively low sensitivity of FAST implies low ability of ERs in detecting abdominal free fluid, indicating a clear need for greater emphasis on education. As a suggestion, carrying out sonography during diagnostic peritoneal lavage (DPL) after introducing the fluid into the abdominal cavity or in patients with confirmed ascites could be beneficial for greater eye familiarity with free fluid appearance in the abdomen during training courses. After training, emergency department residents can perform FAST with high accuracy and specificity, similar to radiology residents, in patients with blunt abdominal trauma.
